# Contemporary Management of Localized Resectable Pancreatic Cancer

**DOI:** 10.3390/cancers10010024

**Published:** 2018-01-20

**Authors:** Anuhya Kommalapati, Sri Harsha Tella, Gaurav Goyal, Wen Wee Ma, Amit Mahipal

**Affiliations:** 1 Department of Internal Medicine, University of South Carolina School of Medicine, Columbia, SC 29209, USA; anuhya781@gmail.com (A.K.); sriharsha.tella@uscmed.sc.edu (S.H.T.); 2 Department of Medical Oncology, Mayo Clinic, Rochester, MN 55902, USA; Goyal.Gaurav@mayo.edu (G.G.); ma.wen@mayo.edu (W.W.M.)

**Keywords:** pancreatic cancer, neoadjuvant therapy, R0 resection, gemcitabine, 5-fluorouracil, FOLFIRNOX

## Abstract

Pancreatic cancer is the third most common cause of cancer deaths in the United States. Surgical resection with negative margins still constitutes the cornerstone of potentially curative therapy, but is possible only in 15–20% of patients at the time of initial diagnosis. Accumulating evidence suggests that the neoadjuvant approach may improve R0 resection rate in localized resectable and borderline resectable diseases, and potentially downstage locally advanced disease to achieve surgical resection, though the impact on survival is to be determined. Despite advancements in the last decade in developing effective combinational chemo-radio therapeutic options, preoperative treatment strategies, and better peri-operative care, pancreatic cancer continues to carry a dismal prognosis in the majority. Prodigious efforts are currently being made in optimizing the neoadjuvant therapy with a better toxicity profile, developing novel agents, imaging techniques, and identification of biomarkers for the disease. Advancement in our understanding of the tumor microenvironment and molecular pathology is urgently needed to facilitate the development of novel targeted and immunotherapies for this setting. In this review, we detail the current literature on contemporary management of resectable, borderline resectable and locally advanced pancreatic cancer with a focus on future directions in the field.

## 1. Epidemiology

Pancreatic cancer is the third leading cause of cancer deaths in the United States (US), with a 5-year survival rate of approximately 7–8% [1]. The estimated incident cases and deaths in the US for the year 2017 are 53,670 and 43,090, accounting for about 3% of cancer diagnoses and 7% of cancer deaths [1]. The dismal prognosis of pancreatic cancer can be attributed to several reasons: initial diagnosis at advanced stages, aggressive nature of the disease with early vascular and neural invasion leading to early distant metastases, resistance to conventional treatment options of chemotherapy and radiation therapy, and presence of multiple genetic/epigenetic alterations and complex tumor microenvironments [2]. Surgical removal of the tumor with negative margins is still the cornerstone of potentially curative therapy, but only 15–20% of patients are eligible at diagnosis. Unresectability is usually due to local vascular invasion (borderline resectable or locally advanced disease) and/or distant metastases. Major improvements in systemic therapy recently significantly enhanced the neoadjuvant treatment approach to managing borderline resectable disease and possibly locally advanced disease [3]. In addition, modern advancements in imaging and surgical techniques have led to a better delineation of resectable disease and decreased post-operative mortality and morbidity, especially in high volume centers [4]. The role of combined-modality therapy with contemporary systemic [5] and surgical therapy for resectable pancreatic cancer is evolving with the recent development and completion of major, multi-institutional clinical trials [6]. In this article, we discuss the key aspects of contemporary management of resectable pancreatic cancer, and provide an outlook of the critical challenges to tackling this deadly disease.

## 2. Staging System: Clinical and TNM

Imaging studies play a key role in the appropriate staging and management of pancreatic cancer [7]. The aim of the staging workup is to delineate the extent of disease spread and to identify patients who are eligible for curative intent surgical resection. Staging is accomplished by pancreas protocol computed tomographic (CT) scan with arterial and venous phase enhancement [8]. Obtaining a thin, preferably submilli­meter sections multidetector CT scan of the chest and abdomen with non-contrast, arterial, pancreatic parenchymal and portal venous phase contrast enhancement should be the first step in the staging of pancreatic cancer. The multiphasic protocol helps us in determining resectability by having a better visualization of surrounding vasculature. This is achieved by selective visualization of arterial (superior mesenteric artery, celiac axis, gastroduodenal artery, and common hepatic artery) and venous (superior mesenteric vein, and portal vein) structures. Pancreatic protocol CT has an excellent sensitivity (89–97%) and negative predictive value. MRI has equal sensitivity in determining tumor resectability and replaces CT scan if patient has contrast allergy [7]. Though CT and magnetic resonance imagining (MRI) provide a detailed picture of local extent of the disease, they are not very sensitive in detecting small hepatic and peritoneal metastases. Positron emission tomography (PET) scan when combined with anatomical imaging can increase the sensitivity of detecting metastatic disease thereby preventing futile surgery [9].

PET-MRI, a relatively newer fusion imaging technique is shown to provide a detailed information on the extent of cancer involvement by detecting the spread of cancer to the main pancreatic duct, collateral veins, superior mesenteric, celiac artery involvement [10,11]. Moreover, PET-MRI is shown to provide a better diagnostic accuracy in detecting liver metastases compared to PET-CT [12]. Endoscopic ultrasound and staging laparoscopy are performed on a case by case basis. 

Endoscopic ultrasound is used in determining portal vein extension, and for confirming histological diagnosis before initiating neoadjuvant therapy. Staging laparoscopy can be considered as an adjunct modality particularly in patients with a high likelihood of occult metastatic disease including tumor located in body or tail, large >3 cm, imaging suggestive of lymph node involvement, very high CA 19-9 [7,8].

Pancreatic cancer can be staged using clinical staging system or American Joint Committee on Cancer/Union for International Cancer Control-Tumor Node Metastasis (AJCC/UICC TNM) staging system. According to National Comprehensive Cancer Network (NCCN) [13], pancreatic cancer is clinically staged into resectable, borderline resectable and unresectable disease based on the extent of the disease. Vascular invasion determines the distinct stages in localized pancreatic cancer. The comparable AJCC/UICC TNM staging [14] with this clinical staging is summarized in Table 1. Clinical staging is more commonly used to determine resectability and management approaches.

## 3. Resectable Pancreatic Disease

Surgical resection is recommended for patients who have localized disease with no metastases, good performance status (based on Eastern Co-operative Oncology group (ECOG) score), no significant comorbidities, and no radiographic interface between the primary tumor and the mesenteric vasculature [8]. Despite advanced surgical techniques and perioperative care, majority of patients who receive surgical therapy die because of local recurrence or distant metastatic disease after a curative-intent surgery [15]. It is of utmost importance that negative margins (R0 resection) are obtained during surgical resection. Patients who have gross disease at surgical margins (R2 resection) do not derive any benefit with surgical resection and the outcomes are similar to the patients who did not undergo surgery. Surgeries performed at high volume centers (>5 surgeries/year) had a better R0 resection rates and a better overall survival [16,17,18]. This is most likely secondary to large experienced comprehensive care team, surgical experience of complicated procedure, better selection of patients and intense hospital monitoring procedures that are available at high volume centers.

It is also important to note that no standard pathological reporting for R0 resection exists, leading to vastly different R0/R1 rates that are being reported by various studies [19,20]. Resection with negative margins is defined as R0 resection by AJCC/UICC, whereas presence of microscopic residual is identified as R1 resection. However, no histologic definitions are specifically mentioned in this classification. In the United States, R1 resection is defined as the presence of microscopic tumor cells on the resected tissue margin (0 mm rule). On the contrary, British Royal College of Pathology (RCPath) considers a resection as R1 resection if the tumor cells are present within 1 mm of the resected margin [19]. This “1 mm” rule reporting by RCPath leads to a 1.3–1.8-fold increase in R1 resection rates compared to that of “0 mm” rule used in the United States [19,21,22]. As the tumor positive margins in pancreatic resection is an important prognostic indicator, a standardized definition of R0/R1 resection is highly warranted to better analyze the results of multi-center/multi-national clinical trials.

Adjuvant chemotherapy is typically recommended for patients undergoing potentially curative resection for pancreatic cancer. A multi-national trial [23,24] showed a better disease-free survival in R0/R1 resection patients who received gemcitabine (1000 mg/m^2^ days 1, 8, and 15 every four weeks for six months) as an adjuvant therapy compared to observation group. A significant improvement in overall survival (OS) was also noted in 5 and 10-year follow up in the adjuvant therapy group (5-year OS: 21 vs. 10 percent; 10-year 12.2 vs. 7.7 percent). Another multinational European study [25] showed similar positive outcomes of improved OS in patients who received 5-Fluorouracil and leucovorin as adjuvant therapy. Patients who completed 6 months course of adjuvant therapy had a better OS compared to their counterparts. Major adjuvant therapy trials are summarized in Table 2 [23,25,26,27,28,29,30,31]. As an international randomized (ESPAC-4) trial [28] showed an OS benefit with a combination of gemcitabine plus capecitabine compared to gemcitabine alone (OS: 28 months vs. 25.5 months; HR for death 0.82, 95% CI 0.68–0.98), the combination therapy is currently recommended as standard of care adjuvant therapy in patients who had surgical resection with or without node positivity. The ongoing clinical trials (NCT02172976 (comparison of Gemcitabine and FOLFIRINOX), NCT01595321 (Nab-paclitaxel and Gemcitabine vs. Gemcitabine Alone)) may provide more information on the benefit of adjuvant chemotherapy in resected pancreatic cancer.

Though adjuvant chemotherapy alone has shown improved OS, the role of radiation therapy in addition to chemotherapy remains controversial. A multi-center European trial (ESPAC-1) [26,32] showed no OS benefit in the group who were treated with adjuvant 5-florouracil and radiation therapy (15.5 months) compared to the group who did not receive the therapy (16.1 months). In fact, a post-study intent-to-treat analysis showed poor OS in the group who received chemoradiation. In contrary, retrospective studies from Johns Hopkins and Mayo Clinic showed positive outcomes of improved survival with adjuvant chemoradiation [33,34]. However, one must be cautious while interpreting the benefits of adjuvant chemoradiation based on the two studies given their retrospective nature. A prospective series [33] involving patients with margin and node positive resections has showed statistically significant improved OS duration (21 vs. 14 months) and 5-year OS (20% vs. 15%) among patients who received adjuvant chemoradiation. Similar encouraging results of OS benefit were seen in R0 resection, high grade and node positive patients who received adjuvant chemoradiation (25 vs. 19 months; 2-year OS 50% vs. 39%) [34]. A matched pair analysis [35] of these two studies showed an OS benefit with adjuvant chemoradiation (RR: 0.59 [0.48–0.72]). Nonetheless, the role of radiation therapy remains controversial and adjuvant chemotherapy is currently recommended for R1 resections. The ongoing clinical trials (NCT01357525, NCT01595321) may provide more information on the benefit of adjuvant radiotherapy in resected pancreatic cancer.

A previous adjuvant therapy (ESPAC-3) [36] trial showed that patients who completed full 6 months of adjuvant therapy with either gemcitabine or 5-FU did have better OS than those who did not (median survival 28 versus 15 months, hazard ratio (HR) for death 0.51, 95% CI 0.44–0.60). Nonetheless, it is estimated that only 50% of patients who had curative-intent surgery receive adjuvant therapy due to issues associated with post-operative complications, recovery and performance status [37]. This opened a new insight of treating resectable disease with neoadjuvant therapy with an idea that chemotherapy or radiation therapy may be better tolerated in patients who have not undergone a major surgical procedure. Patients who develop metastatic disease during neoadjuvant therapy will be spared unnecessary surgery. In addition, neoadjuvant therapy seems to achieve a lower rate of perineural and lympho-vascular infiltration rates as well as higher R0-resection rates in a meta-analysis [38]. Post-operative complications were comparable between patients who did and did not receive neoadjuvant therapy [39]. A retrospective analysis [40] of single institution experience showed that median OS duration was significantly longer in a subset of patients that were younger (age < 50 years), had a baseline serum cancer antigen 19-9 < 200 U/mL and received gemcitabine as a radiosensitizer. Selective neoadjuvant therapy trials and retrospective studies in resectable pancreatic cancer are summarized in Table 3.

Despite the encouraging results from these neoadjuvant trials [41,42,43,44,45] (Table 3), there are several concerns to be considered when recommending neoadjuvant therapy. First, the patients may progress during neoadjuvant treatment to unresectable disease [50], though surgery is unlikely to benefit those with aggressive and/or micrometastatic disease [51]. Second, the putative benefits of neoadjuvant treatment in localized resectable tumors have not been evaluated in randomized controlled trials [52]. Third, the optimal chemotherapy regimens in neoadjuvant therapy has not been determined and varies by treating facility. Furthermore, the role of radiotherapy remains equally controversial. Also, the definition of surgical resectability varied among the studies and was highly dependent on the individual expertise of the facility. Fourth, current understanding of the predictors of treatment response is limited. Regardless, neoadjuvant therapy will be increasingly utilized in the management of localized resectable disease and randomized controlled trial is urgently needed to address these concerns, though will be increasingly difficult to conduct.

## 4. Borderline Resectable Disease

Borderline resectable PC is typically defined as ‘the imprecise continuum between radiologically and technically resectable and unresectable disease’. Table 1 summarizes the different classification systems utilized for defining borderline resectable pancreatic cancer. Though the exact definition is contested [7], common understanding is that tumor down-staging is needed to achieve R0 resection in borderline resectable PC. A prospective study [53] evaluated the safety and efficacy of neoadjuvant therapy with gemcitabine/capecitabine (3–6 cycles of gemcitabine (GEM) + capecitabine every 3 weeks). Eleven out of 18 patients (61%) had surgical resection; and 9/11 (82%) had R0 resection. The median OS in surgery group (23.1 months) was significantly longer compared to that of no-surgery group (13.2 months) (*p* = 0.01). A retrospective analysis from MD Anderson Cancer Center showed 97% R0 resection rates in patients who received induction chemotherapy, chemoradiation or both; the patients who received surgical resection had higher median OS (40 months vs. 21 months) [54]. After neoadjuvant therapy, per multiple retrospective studies, approximately 50% of the patients are able to undergo resection [7]. A multi-institutional prospective study evaluating neoadjuvant concurrent gemcitabine, oxaliplatin and radiation in 39 patients with borderline resectable pancreatic cancer showed that 28 patients had surgical resection with median OS of 25.4 months [55]. About 70% had R0 resection. Similar higher percent of R0 resection rates were also seen in a study [56] that evaluated the combination of gemcitabine and radiation therapy (98% had R0 resection). However, five-year cumulative incidence of peritoneal and distant recurrence was higher in borderline resectable group compared to resectable group.

Another single institutional study evaluated the efficacy and safety of capecitabine based chemoradiation in borderline resectable disease [57]. Forty patients received capecitabine plus external beam radiation in conventional fractionation (50.4 Gy in 28 fractions) or in an accelerated protocol (50 Gy in 20 fractions). About 46% of the patients proceeded to surgery with 96% R0 resection rate. Similar encouraging results were seen in a study from Moffit cancer center where 110 patients received induction chemotherapy (gemcitabine, docetaxel and capecitabine for 3 cycles) followed by stereotactic body radiation therapy (SBRT) and 51% patients were able to proceed to surgery with 96% R0 resection rates [58]. Moreover, a complete pathological response was seen in 7% of patients.

Newer combination therapies, including that combining 5-fluorouracil, folinic acid, oxaliplatin and irinotecan (FOLFIRNOX), and gemcitabine plus nab-paclitaxel have significantly improved the surgical outcome [8]. A prospective, multi-institutional study evaluating neoadjuvant FOLFIRNOX, sequenced with concurrent external beam radiation and capecitabine in borderline resectable disease showed a high resectable percentage (68%) with 93% R0 resection rates [59].

Overall, the results from studies evaluating gemcitabine-based combinations or FOLFIRINOX therapy prior to surgery are encouraging. R0 resection rate in these patients ranged from 38–100% [53,55,56,59,60] (Table 4). However, it is important to note that most of these studies are retrospective in nature and there was no control ‘upfront surgery’ arm. This raises a question of true benefit of neoadjuvant therapy in comparison to ‘upfront surgery followed by adjuvant therapy’.

Due to lack of clear consensus data, patients with the borderline resectable disease should be encouraged to participate in clinical trials (e.g., ESPAC-5, SWOG S1505) [8]. Though concurrent chemoradiotherapy and stereotactic body RT have been utilized in some institutions, the contribution by radiotherapy to successful R0 resection following intensive combination chemotherapy (especially FOLFIRINOX) is unclear.

## 5. Locally Advanced Pancreatic Cancer (LAPC)

The term LAPC implies the involvement of adjacent structures, particularly major arteries, by the pancreas tumor that precludes surgical resection, without evidence of distant metastases [61]. Despite clear guidelines by various professional societies/associations, the presentations of borderline resectable and locally advanced disease can overlap clinically that the assessment/staging becomes subjective and varies among practitioners and institutions. Approximately half of the patients with pancreatic cancer get diagnosed in LAPC stage which curative surgical resection is not feasible [61,62].

Historically, patients with LAPC managed surgically had a relatively poor prognosis (5-year survival rates of 7–25%) due to high rates of margin-positivity (R1 resections) [63]. Recent advancements in multimodality therapies and aggressive surgical approaches by combined vascular resections and reconstruction seem to increase the chance for potentially curative resection [64]. Administration of neoadjuvant therapy to shrink the tumor burden and advanced surgical techniques combined with arterial resection (celiac artery, common hepatic artery and superior mesenteric artery) have been reported with some beneficial effects on the prognosis in select patients [65,66,67]. For example, a single-institution study evaluating neo-adjuvant stereotactic body radiation therapy achieved R0 resection in 84% of patients with LAPC [63]. Longer term neoadjuvant therapy (≥6 months) studies showed more downstaging of the tumor leading to higher resection rates. A retrospective analysis from a single institution showed that prolonged chemotherapy with gemcitabine, 5-FU individually or in combination for a median of 7.1 (5.4–9.6) months showed that about 86% of patients had R0 resections (total *n* = 49; 42 had R0 resection) [68]. It is important to note that this study [68] included both borderline resectable and LAPC.

Similar results were reported in a Japanese prospective trial that evaluated the efficacy of prolonged pre-operative therapy in downstaging of unresectable disease in 34 patients. The treatment approach consisted of 6 months of gemcitabine therapy following an initial chemoradiation with gemcitabine alone or gemcitabine + S-1 [69]. The approach successfully downstaged seven patients (20%) in which four underwent surgical resection.

FOLFIRINOX is becoming the preferred systemic chemotherapy regimen in neoadjuvant management of pancreatic cancer in view of the high response rate observed. A systemic review and meta-analysis of efficacy of FOLFIRINOX (about 57% received combination radiation therapy) in LAPC showed that about 26% (pooled proportion) patients were able to get surgical resection of the tumor and 74% of these patients had R0 resection [70]. Though encouraging, well design clinical trials, preferably randomized, are needed to determine the optimal neoadjuvant approach and long-term outcomes in these patients.

On the contrary, the shrinkage of a radiologically detectable tumor after a downstaging treatment for LAPC is extremely rare [71]. Moreover, given the limitations of currently available imaging modalities, margin assessment and the extent of pathologic response after an intensive systemic therapy remains controversial. The other critical point to consider is whether the regression of tumor size is obtained due to complete tumor regression or if it is just a marker of reduction in density of tumor cells [72]. The latter could potentially lead to R1 resections. Some studies have evaluated the potential role of carbohydrate antigen 19-9 (CA 19-9) to assess the tumor response. Though CA 19-9 is not a valid prognostic marker for survival, retrospective analyses have shown that CA 19-9 levels decrease in patients who responded to neoadjuvant therapy [73,74,75].

Overall, the resection rates of locally advanced disease are increasing especially with prolonged duration of neoadjuvant therapy as seen in recent studies [68,69]. It is important to note that the benefits of curative surgical procedure in LAPC are based only on small sample sized retrospective analyses and more prospective randomized trials are needed to provide the bases for the surgical approach for LAPC. 

## 6. Conclusions and Future Directions

Despite the development of highly active systemic chemo-regimens, preoperative treatment strategies and better peri-operative care, the management of localized pancreatic cancer remains highly challenging. Contemporary pancreatic cancer drug therapy centers on combining multiple cytotoxic agents with overlapping dose limiting toxicities, and there is an urgent need to develop novel approaches such as molecularly-targeted and immuno-therapeutics that circumvent these limitations. However, a major challenge for developing molecularly-targeted therapies is the presence of few prevalent genetic mutations- KRAS (activating), CDKN2A (encoding p16), TP53 and SMAD4 (inactivating) and none of these are currently druggable [2]. To overcome this, further studies are to be focused on identifying key multimodality-signaling targets. Pancreatic cancer is a heterogenous disease with multiple exomic genetic alterations in about 12 key cellular signaling pathways (e.g., Poly (ADP-ribose) polymerase inhibition, CD40 activation, hedgehog signaling inhibition, and RET inhibition) [76,77,78,79,80]. The investigation of vulnerable genes in these altered signaling pathways can contribute to development of newer targeted drug therapies. Certain animal studies that tested therapeutic targeting have shown promising results thus far [78,80,81]. Indeed, some ongoing clinical trials are investigating the potential of targeted, and immune therapies in the management of pancreatic cancer [2,82,83].

Other key areas that will improve the outcome of localized pancreatic cancer include early detection, standardization of care and improving surgical outcomes. Early detection of disease can be obtained by identifying the population at risk and developing better imaging modalities that can identify the cancer at an early stage. This can be achieved by the use of novel biomarkers (C4b-binding protein α-chain (C4BPA), CA 19-9, Plectin-1), imaging strategies such as endoscopic ultrasound, MRI, CT for early detection [84,85]. So far, all these strategies evaluated for early detection of disease are from single institution studies and are of a relatively small sample size. Further analysis in large well-randomized cohort studies will hopefully help us in identifying the predictive markers for early detection of the disease. Furthermore, the current proteomic approaches suffer from limited sensitivity and specificity. Additional serum biomarkers with high sensitivity and specificity are needed to improve the early detection rates of the disease. Standardization of care across the institutions can be achieved by having unified definitions with internationally acceptable standards so that clinical trials can be compared and analyzed in a better way. Better surgical outcomes can be obtained from a combination of factors such as high surgical volume, advanced technology and individualization of care. This can be achieved by managing patients with localized pancreatic cancer at dedicated centers with experienced multi-disciplinary team.

## Figures and Tables

**Table 1:** AJCC/UICC-TNM and clinical staging.

TNM Staging	Clinical Staging	NCCN Criteria of Defining Resectability
0 Tis N_0_ M_0_	Resectable	Resectable: No extension into regional arteries (CA, CHA, SMA) or veins (SMV, PV), PV extension to < 180°
IA T_1_ N_0_ M_0_	Resectable	
IB T_2_ N_0_ M_0_	Resectable	
IIA T_3_ N_0_ M_0_	Resectable	
IIB T_1_ N_1_ M_1_ T_2_ N_1_ M_0_ T_3_ N_1_ M_0_	Resectable	
III T_4_ anyN M_0_	Borderline resectable/ locally advanced/ unresectable	Borderline resectable: Solid tumor contact with -CHA/SMA ≤ 180° -CA ≤ 180° or > 180° with inert aorta and gastroduodenal artery. -SMV/PV > 180° or ≤ 180° with contour irregularity/thrombosis permitting safe reconstruction Or -IVC involvement Unresectable: Solid tumor contact with -CA/SMA > 180° or -CA and aorta engrossment -1st jejunal branch of SMA -Proximal jejunal draining into SMV Or -Tumor involvement or occlusion of SMV/PV rendering it unreconstructible
IV AnyT anyN M_1_	Unresectable	

CA: Celiac artery; CHA: Common hepatic artery; PV: Portal vein; SMA: Superior mesenteric artery; SMV: Superior mesenteric vein.

**Table 2:** Selective prospective adjuvant therapy trials in resectable pancreatic cancer.

Trial	Regimen	Significance/Outcome	Comments
CONKO-001 [23] (*n* = 368)	GEM (1 g/m^2^ days 1, 8, 15 every 4 weeks) vs. observation for 6 months	DFS: 13.4 (GEM) vs. 6.7 months (obs) Five-year OS: 20.7% (GEM) vs. 10.4% (Obs) Ten-year OS: 12.2% (GEM) vs. 7.7% (Obs)	Phase 1b trial
ESPAC-1 [26] (*n* = 289)	CT with 5-FU + RT vs. CT only and then continue with CT vs. CT vs. Obs	Median OS: 21.6 months Chemotherapy based on 5-FU No survival benefit in CT + RT group.	Significant benefit in 2-and 5-year ITT in CT group
ESPAC-3 [25] (*n* = 1088)	GEM (1 g/m^2^ days 1,8, 15 every 4 weeks) vs. 5-FU/LV (20 mg/m^2^ iv bolus followed by 5-FU, 425 mg/m^2^ iv bolus days 1–5 of every 28) for 6 months	Median OS: 23.6 months (GEM) vs. 23 months (5-FU/LV)	Grade-3 and 4 toxicities and more hospitalizations in 5-FU/LV group
JASPAC-01 [27] (*n* = 385)	Gemcitabine or S-1	S-1 non-inferior to GEM, lower mortality rate with S-1 (HR: 0.57, 95% CI 0.44–0.72, five-year survival 44 vs. 24%).	S-1 is available only in Japan. GEM cohort had more leukopenia and somatitis
ESPAC-4 [28] (*n* = 732)	GEM vs. GEM-capecitabine	Median OS: 28 months (GEM-capecitabine) vs. 25.5 months (GEM)	60% of patients in each arm had R1 resection; 80% had node positivity.

GEM: Gemcitabine; DFS: Disease Free Survival; obs: Observation; CT: Chemotherapy; 5-FU: 5- Fluorouracil; RT: Radiation therapy; LV: Leucovorin; iv: intravenous; S-1: S-1 is an oral fluoropyrimidine that includes ftorafur (tegafur), gimeracil (5-chloro-2,4 dihydropyridine), and oteracil (potassium oxonate).

**Table 3:** Selective neoadjuvant therapy trials and retrospective studies in resectable pancreatic cancer.

Trial	Regimen	Significance/Outcome	Comments
Palmer et al. [41] (*n* = 50)	GEM (1000 mg/m^2^) vs. GEM + Cisplatin (25 mg/m^2^)	27 (54%) underwent pancreatic resection, 9 (38%) in the gemcitabine arm & 18 (70%) in the combination arm	No increased surgical complications noted in either arm. Grade 3/4 toxicity same in both the arms.
Varadhachary et al. [42] (*n* = 90)	GEM/cisplatin followed by GEM-based chemoradiation	79 (88%) completed chemoradiotherapy. 51 (66%) had pancreatic resection. Median OS of surgery group: 31 months; Median OS for non-surgery group: 10.5 months (*p* < 0.001)	No tumor progression was noted during the chemoradiotherapy
Sho, M. et al. [43] (*n* = 132)	GEM (1000 mg/m^2^) + Radiation (50–54 Gy) vs. no therapy	GEM + Rad group: Median survival: 28 months. 92% had R0 resections.	Post-operative adjuvant therapy delayed in GEM+Rad group due to poor nutritional status.
O’Reilly et al. [44] (*n* = 38)	GEM (1000 mg/m^2^) + Oxaliplatin (80 mg/m^2^)	Thirty-five (92%) completed therapy. Twenty-seven (71%) patients underwent tumor resection; 26 initiated adjuvant therapy and 23 (60.5%) completed. The 18-month survival was 63% (24 patients alive). Median OS (for 38 patients): 27.2 months Median DSS: 30.6 months	Single-arm non-randomized trial.
Golcher et al. [45] (*n* = 73)	GEM/Cisplatin + radiation; Patients randomized to neoadjuvant therapy vs no neoadjuvant therapy	No difference in R0, N0 resection rates, post-operative complications and median OS (*p* = 0.79)	First randomized prospective trial.
White et al. [46] (*n* = 82)	5-flourouracil (5FU)-based chemotherapy	Despite more locally advanced tumors in neoadjuvant therapy cohort (21 vs. 8% (*p* < 0.05)), neoadjuvant therapy cohort had smaller resected tumors, less likely to have T3 tumors and fewer positive lymph nodes (*p* < 0.01)	Retrospective single center analysis
Papelezova et al. [47] (*n* = 236)	Total dose of 4500 cGy of external beam radiation therapy followed by 5-fluorouracil (5-FU)-based chemotherapy (oral capecitabine or infusional 5-FU) at radio sensitizing doses	Median overall survival (OS) i was higher in neoadjuvant therapy cohort (27 months vs. 17 months) (*p* = 0.04) Lower incidence of positive lymph nodes and smaller tumor size noted in neoadjuvant therapy cohort	Retrospective single center analysis. Neoadjuvant therapy is termed as “preop chemotherapy” in this analysis
Artinyan et al. [48], (total *n* = 458, neoadjuvant therapy *n* = 39)	NA	Lower rate of lymph node positivity in the neoadjuvant group (45% vs. 65%; *p* = 0.011) despite a higher rate of extra-pancreatic tumor extension. Better OS in neoadjuvant therapy group (median survival, 34 vs. 19 months; *p* = 0.003)	Population based study from California cancer surveillance program
Cooper et al. [49] (*n* = 179)	External-beam radiation (typically to 30 Gy in 10 fractions or 50.4 Gy in 28 fractions) with concurrent GEM, 5-FU, capecitabine or FOLFIRINOX * Systemic chemotherapy with GEM ± cisplatin/erlotinib was delivered before chemoradiation in selected patients.	Median OS of all patients with neoadjuvant therapy (16.6; 2.1 to 142.7 months) was similar to up-front resection (15.1; 5.4 to 100.8) months (*p* = 0.53)	Retrospective single center analysis in elderly patients.

GEM: Gemcitabine; * (85 mg/m^2^ of oxaliplatin, 180 mg/m^2^ of irinotecan hydrochloride, 400 mg/m^2^ of leucovorin calcium, and then 2400 mg/m^2^ of 5-fluorouracil for 4 cycles); 5-FU: 5-Fluorouracil; NA: Data not available.

**Table 4:** Selective neoadjuvant therapy trials in borderline-resectable pancreatic cancer.

Trial	Regimen	Significance/Outcome	Comments
Lee, J. et al. [53] (*n* = 18)	3–6 cycles of gemcitabine (GEM) + capecitabine every 3 weeks	Eleven (61%) had surgical resection; 82% had R0 resection. Median OS in surgery group: 23.1 months Median OS in no-surgery group: 13.2 months (*p* = 0.017)	43-month follow-up: Median progression-free survival of 10.0 months
Kim, E.J. et al. [55] (*n* = 39)	Two 28-day cycles of GEM (1 g/m^2^ over 30 min on days 1, 8, and 15) and oxaliplatin (85 mg/m^2^ on days 1 and 15) with RT during cycle 1 (30 Gray (Gy) in 2-Gy fractions)	Twenty-eight had surgical resection; 70% had R0 resection Median survival of borderline resectable: 18.4 months (95% CI, 11–27.1); resectable disease: 26.5 months (95% CI, 11.8–44.7)	28 had surgical resection with median survival of 25.4 months.
Motoi, F. et al. [60] (*n* = 16)	GEM + S-1	Two-year survival: 31.5% Median OS in surgery group: 34.7 months. Surgery group had better survival (*p* = 0.0017)	Neutropenia in treatment group.
Tahahashi. H. [56] (*n* = 80)	GEM-Radiotherapy	Forty-three (54%) had surgical resection; Forty-two (98%) had R0 resection. Five-year cumulative incidence of peritoneal and distant recurrence higher in borderline resectable group compared to resectable group	5-year survival: 34% (less than resectable group-57%) (*p* = 0.02)
Katz, M. et al. [59] (*n* = 22)	FOLFIRINOX * then chemoradiotherapy	Fifteen (68%) had surgical resection; 14 (93%) had R0 resection.	Median OS: 21.7 months

GEM: Gemcitabine; * (85 mg/m^2^ of oxaliplatin, 180 mg/m^2^ of irinotecan hydrochloride, 400 mg/m^2^ of leucovorin calcium, and then 2400 mg/m^2^ of 5-fluorouracil for 4 cycles) followed by 5.5 weeks of external-beam radiation (50.4 Gy delivered in 28 daily fractions) with capecitabine (825 mg/m^2^ orally twice daily).
